# Transforming Tea Catechins into Potent Anticancer Compound: Analysis of Three Boronated-PEG Delivery System

**DOI:** 10.3390/mi13010045

**Published:** 2021-12-28

**Authors:** Mingyan Guo, Lukas Marek, Yixia Liang, Phei Er Saw

**Affiliations:** 1 Guangdong Provincial Key Laboratory of Malignant Tumor Epigenetics and Gene Regulation, Sun Yat-sen Memorial Hospital, Sun Yat-sen University, Guangzhou 510120, China; guomyan@mail.sysu.edu.cn (M.G.); liangyx65@mail2.sysu.edu.cn (Y.L.); 2 Medical Research Center, Sun Yat-sen Memorial Hospital, Sun Yat-sen University, Guangzhou 510120, China; 3 Department of Anesthesiology, Sun Yat-sen Memorial Hospital, Sun Yat-sen University, Guangzhou 510120, China; 4 Faculty of Engineering, Nanotechnology Engineering, University of Waterloo, Waterloo, ON N2L 3G1, Canada; lukasmarek@hotmail.com

**Keywords:** tea catechin, boronated-PEG, piceatannol, epigallocatechin gallate hydrophilic, l-epicatechin

## Abstract

Chemotherapy has led to many undesirable side effects, as these are toxic drugs that are unable to differentiate between cancer and normal cells. Polyphenols (tea catechins) are an ideal option as alternative chemotherapeutics owing to their inherent anticancer properties, antioxidant properties and being naturally occurring compounds, are deemed safe for consumption. However, without proper administration, the bioavailability of these compounds is low and inefficient. Therefore, proper delivery of these phenolic compounds is vital for cancer therapy. Herein, we analyzed three potential solutions to creating nanoparticle drugs using naturally occurring phenolic compounds (piceatannol (PIC), epigallocatechin gallate hydrophilic (EGCG) and l-epicatechin (EPI)). By using a simple pi-pi stacking mechanism, we utilized boronated PEG (PEG-Br) as an anchor to efficiently load EPI, PIC and EGCG, respectively, to produce three effective phenolic compound-based nanoparticles, which could be delivered safely in systemic circulation, yet detach from its cargo intracellularly to exert its anticancer effect for effective cancer therapy.

## 1. Introduction

Cancer is a worldwide disease that has millions of sufferers, yearly. There are so many variations of cancer and with that in mind, there are multiple options for treating cancer patients depending on what form of cancer they have. These cancer cells can be killed using a variety of drugs instead of invasive surgical removal, however, each having their negative impact on the human body. In order to find a viable treatment, a drug must have the ability to kill cancer cells, but at the same time not cause the death of nearby healthy cells or minimally impact neighboring cells and systems.

Nanomedicines have become a recent industry, expanding to provide new cutting-edge procedures using nanoscale drugs and delivery systems to combat all sorts of disease, including cancer [1,2,3]. As a result, nanomedicines have been continually evolving and have begun to show promise in curing cancer. However, just like regular drugs, these nanodrugs have toxic effects on both cancer cells and healthy neighboring cells in the human body. Once the drug is administered, it is important for the drug to accumulate in the tumor site and mainly target cancer cells. Therefore, the drug must be stable in the body and also consistent in nature. Through the aid of a nanocarrier, a drug can be safely administered, with long systemic circulation; which could effectively increase drug bioavailability while reducing its systemic toxicity [4]. Additionally, using a naturally occurring phenolic compound may have a less negative toxic effect on healthy cells and as a result the entire body, but with the difficulty of forming a stable nanoparticle that can still administer the contents of the nanoparticle [5,6].

Catechins are natural polyphenolic compounds found in a wide variety of fruits, vegetables and plant-based food and beverages. Green tea extract is a recognized rich dietary source of catechins [7,8]. A broad range of pharmacological activities have been reported for catechins, including antiviral, nephroprotective and anticancer activities, which are strongly associated with their anti-inflammatory, antioxidant and cytotoxic properties [9,10,11]. A recent study also shows that catechins may serve as promising therapeutics in COVID-19-associated AKI due to their well-recognized anti-SARS-CoV-2, and antioxidant and anti-inflammatory properties that mediate their reno-protective activities [12]. Furthermore, compared to drugs, the cost of procuring phenolic compounds is relatively cheaper and cost-effective. In this study, we focus on the possibility that phenolic compounds offer a naturally occurring compound found in teas to be used as a cancer drug treatment therapy, due to its ability to self-assemble and its cytotoxic properties.

L-epicatechin (EPI), (-)-epigallocatechin gallate (EGCG), and piceatannol (PIC) are the three phenolic compounds analyzed in section [7]. Each solution is a phenolic compound and has hydroxyl groups attached to the benzene rings in the compound which result in strong intermolecular interactions due to hydrogen bonding [5,13]. The three solutions are also made up of at least one benzene ring and also have intermolecular interactions through pi stacking. These intermolecular interactions are the driving force for each solution’s self-assembly process in the nanoparticle synthesis. Each solution was also conjugated with polyethylene glycol-boronic acid (PEG-Br) to bind to the hydroxyl groups and form the shell of the nanoparticle. These intermolecular interactions and the conjugation of PEG-Br resulted in a stable self-assembling nanoparticle.

In this work, we looked and analyzed three potential solutions to create nanoparticle drugs using natural occurring phenolic compounds. The three solutions that matched the company requirements were piceatannol (PIC), epigallocatechin gallate hydrophilic (EGCG) and l-epicatechin (EPI). Each solution uses a naturally occurring phenolic compound found in tea to create nanoparticles. Since each solution met the criteria of being a naturally occurring compound that had hydroxyl groups found around the benzene ring, the main concerns were the nanoparticle formation, cell toxicity and economic impact.

## 2. Materials and Methods

### 2.1. Materials

Piceatannol (PIC), epigallocatechin gallate hydrophilic (EGCG) and l-epicatechin (EPI) were purchased from Sigma Aldrich (St. Louis, MO, USA). Boronic acid bonded to PEG (PEG-BR) was a kind gift from the lab of Prof Jun Wu of Sun Yat-sen University. Rhodamine was purchased from Sigma Aldrich (St. Louis, MO, USA).

### 2.2. Polyphenol Nanoparticles (NPs) Synthesis

To prepare EPI-, PIC- and EGCG-encapsulated NPs, we first weighed out 20 mg of PEG-Br and 2 mg of EPI, PIC and EGCG, respectively. Added in 2 mg of EPI, PIC and EGCG into respective vials of PEG-Br before adding 1 mL of HyPure water into the vial with constant stirring. The solution was then passed through 500 µm cellulose membrane syringe filter to obtain polyphenol-encapsulated NPs.

### 2.3. Cell Culture

BT474 and 4T1 cell lines were purchased from ATCC (Washington, DC, USA). The cells were maintained at 37 °C in a humidified cell culture chamber equipped with 5% CO_2_. Cells were maintained in DMEM, 1640 or ECM medium supplemented with 10% or 5% FBS, 100 U/mL penicillin, and 100 µg/mL streptomycin.

### 2.4. In Vitro Cellular Uptake

To visualize the in vitro delivery ability of the NPs, Rhodamine was encapsulated into NP(EPI), NP(PIC) and NP(EGCG) respectively. BT474 cells were grown to ~70% confluence on glass bottom cell culture dishes (⌀ 15 mm; Nest, Wuxi, China). Respective NPs were added to the culture medium at 1 mg/mL concentration. After 2 h incubation at 37 °C, cells were washed three times with PBS and fixed with 4% (*w*/*v*) paraformaldehyde (PFA). Dish bottom with fixed cells were mounted with Dako mounting media and examined using an Olympus Fluoview 1000 confocal microscope (Olympus Imaging Co., Tokyo, Japan).

### 2.5. In Vitro Cell Cytotoxicity

To determine the cellular toxicity of the respective NPs, PEG-Br (negative control), polyphenols (EGCG, PIC, and EPI), and NP(EPI), NP(PIC) and NP(EGCG) were added, respectively, to BT474 cell lines at 5000 cells/well at an increasing concentration of 0.2–2 mg/mL. After 48 h, cells were washed thrice with PBS before cell viability was detected via Alamar blue assay.

### 2.6. Dynamic Light Scattering (DLS) Analysis of Polyphenol NPs

The size distribution and zeta potential of NPs were determined by dynamic light scattering (DLS) analysis using Malvern Panalytical, MA, USA. The data for each sample were obtained from three replicates.

### 2.7. Transmission Electron Microscopy (TEM) Analysis

Samples of 10 µL aliquots of each NP encapsulating EPI, PIC and EGCG were respectively dropped onto a TEM grade carbon-only mesh copper grid. Particles were left on grid in room temperature for 5 min. Each grid was washed five times with distilled water and air-dried. The specimens were visualized using a TECNAI F20 electron microscope (Philips Electronic Instruments Corp., Mahwah, NJ, USA).

### 2.8. Tumor Retention Properties of Polyphenol NPs In Vivo

To visualize the in vivo tumor retention ability of all polyphenol NPs, NP(EPI), NP(PIC) and NP(EGCG) were injected (intravenous injection; i.v.) into 4T1 allograft tumor-bearing mice when the tumor size was around 100 mm^3^. The injection dosage was determined by the dosage of Rhodamine as 10 nmol. After 24 h, the tumor area was observed by live IVIS Imaging system (Perkin Elmer, Wellesley, MA, USA). The laboratory animal facility has been accredited by Association for Assessment and Accreditation of Laboratory Animal Care International (AAALAC), and the Institutional Animal Care and Use Committee (IACUC) of Sun Yat-sen University approved the animal protocol used in this study.

## 3. Results

### 3.1. The Nanoparticle Forming Abilities of EPI, PIC and EGCG Nanoparticles

**Figure 1:**
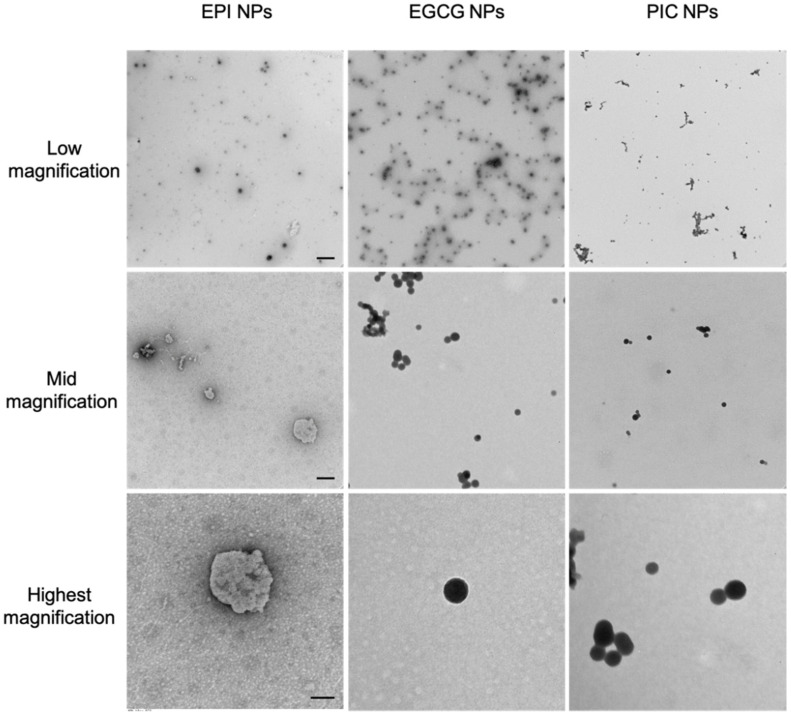
Low, middle and high magnification of TEM analysis revealed a rough diameter for NP(EPI), and spherical uniform particles could be seen in NP(EGCG) and NP(PIC) group.

To determine the nanoparticle forming ability of the polyphenol-encapsulated NPs, we carried out DLS analysis. As seen in Table 1, although all the NPs showed uniform distribution, NP(PIC) had the smallest mean of 301.9 nm, followed by NP(EPI) at 425.2 nm and NP(EGCG) at 465 nm. All three polyphenol NPs showed a bigger diameter (>250 nm). This could be due to the fact that the pi-pi stacking of PEG-Br is transient, with weaker intramolecular bonding between the anchor (PEG-Br) and the cargo (polyphenols). From the TEM analysis seen in Figure 1, NP(EPI) showed slightly roughened edges with a non-symmetrical spherical form; while the other two NP(PIC) and NP(EGCG) showed a smooth spherical shape with uniform distribution in all the TEM magnification, indicating a smoother process of NP development.

### 3.2. The In Vitro Cytotoxicity of PEG-Br, EPI, PIC, EGCG and Their Nanoparticles

**Figure 2:**
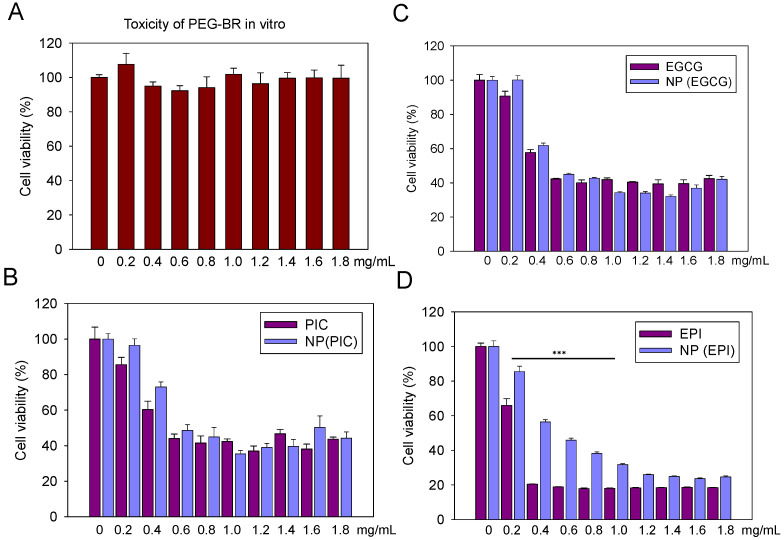
In vitro cytotoxicity analysis revealed that PEG-Br was non-toxic (**A**). EGCG (**B**) and PIC (**C**) retained similar cytotoxicity while EPI (**D**) showed decreased toxicity after encapsulation into NPs.

We used the BT474 cells in vitro to test the in vitro cytotoxicity of PEG-Br, EPI, PIC and EGCG and their NP counterparts. As seen in Figure 2A, PEG-Br did not show any appreciable toxicity indicating its safety to be used as a delivery vehicle. As illustrated in Figure 2B–D, the dose-dependent toxicity of the polyphenols and their NP-encapsulated counterparts are shown. PIC and EGCG both indicated similar cytotoxicity compared to their NP-encapsulated counterparts; while NP(EPI) showed decreased toxicity compared to its parental EPI, especially at a lower dose treatment. However, this phenomenon was corrected at EPI dosage higher than 1.2 mg/mL. This could be attributed to the different nanoparticle-forming abilities of each polyphenol which could determine their cellular uptake efficacy.

### 3.3. The In Vitro Cellular Uptake of EPI, PIC and EGCG Nanoparticles

**Figure 3:**
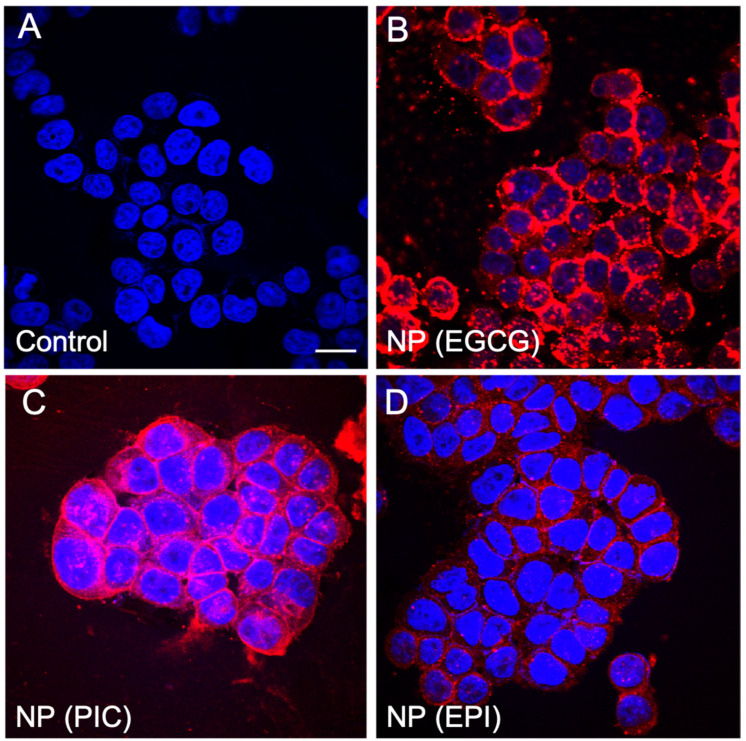
Cellular uptake of control (**A**), rhodamine encapsulated NP(EGCG) (**B**); NP (PIC) (**C**); and NP(EPI) (**D**) in BT474 cells in vitro.

To verify in vitro cellular uptake of EPI, PIC and EGCG nanoparticles, we encapsulated rhodamine in each NP system. After co-incubation for 2 h with BT474 cells, we can clearly see that the highest uptake could be seen in the NP(EGCG) group (Figure 3B), followed by the NP(PIC) (Figure 3C) group while NP(EPI) showed a significantly reduced fluorescence intensity compared to the former two groups (Figure 3D). Therefore, it is clear that the cellular uptake of NP(EPI) was the lowest compared to NP(PIC) and NP(EGCG), indicating that the physical morphology and stability of a nanoparticle is important for its utilization in vitro and in vivo.

### 3.4. The In Vivo Tumoral Uptake of EPI, PIC and EGCG Nanoparticles

**Figure 4:**
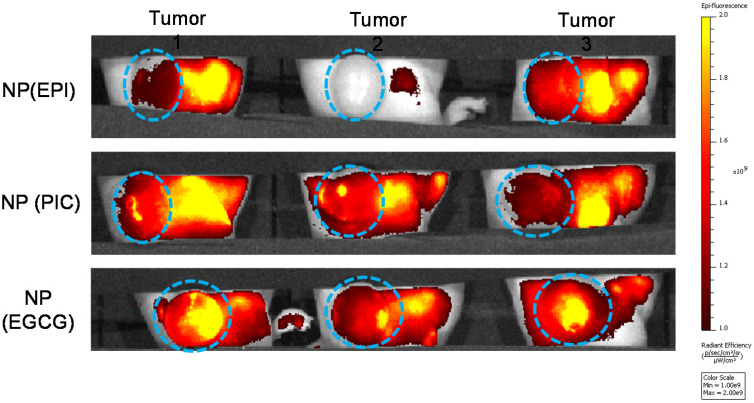
In vivo tumoral uptake of NP(EPI), NP(PIC) and NP(EGCG) in breast cancer allograft model. Blue circle indicated the location of tumor.

To examine the in vivo tumoral uptake of these polyphenol NPs, we used rhodamine-encapsulated NP(PIC), NP(EGCG) and NP(EPI) to be administered through the tail vein into 4T1-tumor bearing mice. After 24 h, live IVIS imaging indicated that NP(EPI) had the lowest uptake of NPs, followed by NP(PIC) while NP(EGCG) had the highest tumor retention (Figure 4). Therefore, we speculate that EGCG might form the most stable NPs with PEG-Br to be delivered efficiently into cells, while being stable in the systemic circulation after i.v. injection.

## 4. Discussion

The main requirement for any cancer therapy is to kill cancer cells, but many other optional requirements must be met as well, in order to have a viable cancer therapy that can continually be used for a wide variety of patients and cancers. Targeting cancer specifically is definitely a requirement that should be met in cancer therapies [14]. However, this does not mean that all of the drugs must accumulate at the cancer site. Simply having a slightly targeted system that would increase the accumulation of the drug at the cancer site compared to chemotherapy would prove to be beneficial and required. A nanoparticle system should be stable, have the ability to carry large loads of cargo, and most importantly, could be accumulated to the tumor site through an enhanced permeability and retention (EPR) effect [5,9,15]. The drug must maintain its toxic effects and as a result should be stable in the sense that it does not break open, releasing its contents and should only do so at the cancer site. The final major requirement for a cancer therapy is that the drug or therapy has proven cytotoxic effects [16]. Therefore, we synthesized the EPI, PIC and EGCG nanoparticles, followed by testing each property, trying to find out the best option for an anticancer nanodrug.

**Figure 5:**
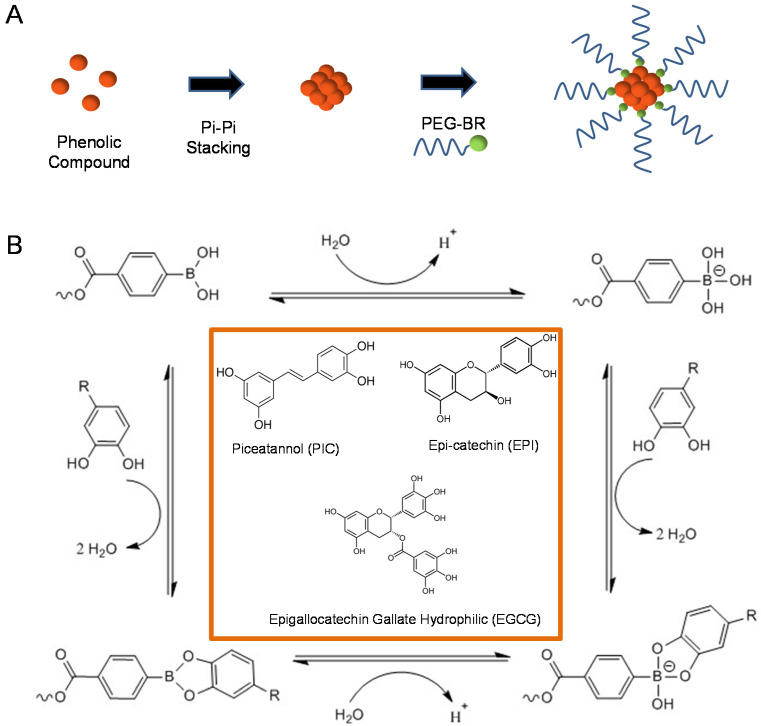
The schematic diagram of the synthesis of polyphenol NPs. (**A**) Schematic representation of the utilization of pi-pi stacking of PEG-Br in the chemical bonding with all phenolic compounds. (**B**) The detailed mechanism of the pi-pi stacking chemistry with PIC, EPI and EGCG structure in the orange box.

The assembly of the drug should be through a self-assembly process where the mixing of two solutions should chemically interact to self-assemble into nanoparticles [17]. For phenolic compounds specifically, the phenolic compounds must have strong intermolecular interactions with each other in a solution of water, such as pi-pi stacking, and should be able to bond to a long-chained compound such as PEG-Br to form its shell as depicted in Figure 5A [18]. Phenolic compounds have been used to treat a select few cancers such as skin cancer and our team sees them as a potentially viable solution that could match all or most of the requirements stated above. L-epicatechin (EPI), (-)-epigallocatechin gallate (EGCG), and piceatannol (PIC) are the three phenolic compounds which were analyzed in the section. Each solution is a phenolic compound and has hydroxyl groups attached to the benzene rings in the compound which result in strong intermolecular interactions due to hydrogen bonding. The three solutions are also made up of at least one benzene ring and also have intermolecular interactions through pi stacking. These intermolecular interactions are the driving force for each solution’s self-assembly process in nanoparticle synthesis [19]. Each solution is also conjugated with polyethylene glycol-boronic acid (PEG-Br) to bind to the hydroxyl groups and form the shell of the nanoparticle (Figure 5B).

We carried out DLS analysis to determine the nanoparticle-forming ability of the polyphenol-encapsulated NPs and found that NP(PIC) had the smallest size, followed by NP(EPI) and NP(EGCG). Compared to conventional nanoprecipitation method [20,21,22], all three of our polyphenol NPs showed bigger diameter (>250 nm). This could be due to the fact that the pi-pi stacking of PEG-Br is transient, with weaker intramolecular bonding between the anchor (PEG-Br) and the cargo (polyphenols). Interestingly, NP(EPI) showed slightly roughened edges with a nonsymmetrical spherical form; indicating that NP(EPI) might not be at its lowest level of entropy during the formation of the NPs. The other two NP(PIC) and NP(EGCG) showed a smooth spherical shape with uniform distribution in all the TEM magnification, indicating a smoother process of NP development. EPI was unable to produce any clear shape of nanoparticles and as a result did not have the stability of a nanoparticle. PIC, on the other hand, did not make very constant rod-shaped nanoparticles, but compared to the spherical nanoparticles of EGCG, it was clear that the rod shape lacked the stability of a spherical nanoparticle, giving EGCG a distinct stability that was far greater than PIC and, especially, EPI.

Then, we tested the in vitro cytotoxicity of PEG-Br, EPI, PIC and EGCG and their NP counterparts in BT474 cells in vitro. We found that PEG-Br did not show any appreciable toxicity, indicating its safety to be used as a delivery vehicle. However, as illustrated in Figure 2B–D, EPI, PIC and EGCG and their NP-encapsulated counterparts showed a dose-dependent toxicity. PIC and EGCG both indicated similar cytotoxicity compared to their NP-encapsulated counterparts, indicating that these polyphenols did not lose their bioactivity after NP encapsulation. Interestingly, NP(EPI) showed decreased toxicity compared to its parental EPI, especially at a lower dose treatment. Relating the cytotoxic benefits of each solution, it was seen that each had their own benefits. EPI and PIC have been researched to be angiogenetic compounds which could slow the growth of cancer [23,24]. This property is beneficial for a drug, but the drug’s main property must be to kill cancer cells and based on the cytotoxic graphs based on the concentrations of phenolic compound introduced to the in vitro environment [25], it was seen that EPI showed no clear cytotoxic signs, while the other two compounds, EGCG and PIC, both had clear ability to kill cancer cells efficiently. This inability to display any visible cytotoxic effects means that once in the body and especially the cancer site, EPI will not be able to effectively kill a rapidly multiplying tumor where cells grow much faster than the majority of the other cells in the human body. This could be attributed to the different nanoparticle-forming abilities of each polyphenol which could determine their cellular uptake efficacy. As shown above, for EPI, the TEM images showed no clear nanoparticle formation and the few nanoparticles visible were irregularly shaped and not stable. The self-assembly formulation, therefore, does not result in proper nanoparticle formation and cannot be injected into a person as a stable nanoparticle.

To verify the above hypothesis, we encapsulated rhodamine in each NP system and carried out in vitro cellular uptake. The results showed that the highest uptake could be seen in the NP(EGCG) group, followed by the NP(PIC) group while NP(EPI) showed significantly reduced fluorescence intensity compared to the former two groups. Therefore, it was clear that the cellular uptake of NP(EPI) was the lowest compared to NP(PIC) and NP(EGCG), indicating that the physical morphology and stability of a nanoparticle is important for its utilization in vitro and in vivo, which was further proved by in vivo study. We used rhodamine-encapsulated NP(PIC), NP(EGCG) and NP(EPI) to be administered through the tail vein into 4T1-tumor bearing mice and the results also showed that NP(EPI) had the lowest uptake of NPs, followed by NP(PIC) while NP(EGCG) had the highest tumor retention. Therefore, we speculate that EGCG might form the most stable NPs with PEG-Br to be delivered efficiently into cells, while being stable in the systemic circulation after i.v. injection. The dermally-applied EGCG creams have been shown to have cancer healing properties, indicating the potential of EGCG as a cancer therapy [26]. The added engineering of EGCG as a nanoparticle allows us to introduce it systematically into an in vivo system, and most likely reach the tumor site through an enhanced permeability and retention (EPR) effect [27]. Since most mouse tumor models exhibited EPR effects, the nanoparticles could therefore be accumulated in the tumor vicinity and accumulate in the tumor region [28].

Various retrospective analyses have determined the high-cost of current chemotherapeutic regimes. For example, the cost for Herceptin intravenous powder for injection 150 mg is around USD 1636 for a supply of one injection. For long-term treatment, this choice might not be viable to many patients. In Sigma Aldrich, the procurement of EPI is USD 50/gram, pure PIC is USD 150/gram, and pure EGCG is USD 150/gram. All these polyphenols are almost a thousand-fold cheaper than current chemotherapeutic drugs. Therefore, given a choice, patients could choose a lower-priced regimen, which is still a beneficial and effective cancer therapeutic.

## 5. Conclusions

By using a simple pi-pi stacking mechanism, we developed three polyphenol-based nanoparticles with good cellular uptake, inherent anticancer properties and good tumor retention in vivo. We also concluded that NP(EGCG) showed the best tumoral uptake and is worthy of further investigation. This simple yet robust method could be used to synthesize other similar phenol-based nanoparticles.

## Figures and Tables

**Table 1:** DLS analysis of EPI, PIC and EGCG NPs.

NP	Run1 (nm)	Run2 (nm)	Run3 (nm)	Mean (nm)
**EPI**	400	458	416.7	425.2
**EGCG**	473.5	464.9	456.5	465
**PIC**	302.5	306.1	297.1	301.9

**Note:** DLS analysis indicated symmetrical distribution of EPI, PIC and EGCG NPs, with an average size of 300~450 nm. DLS: dynamic light scattering.

## Data Availability

All data is available upon request from the corresponding author.
